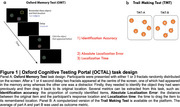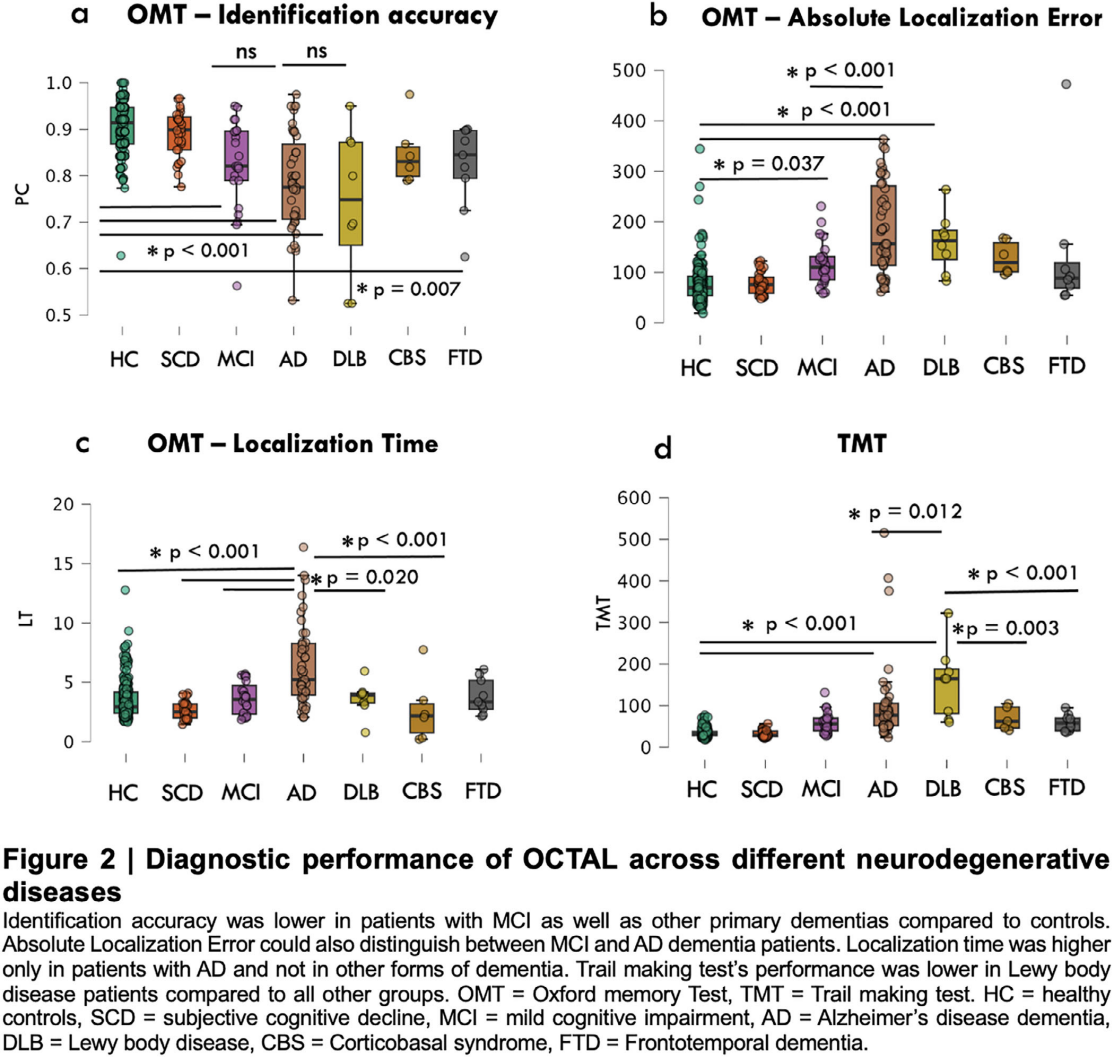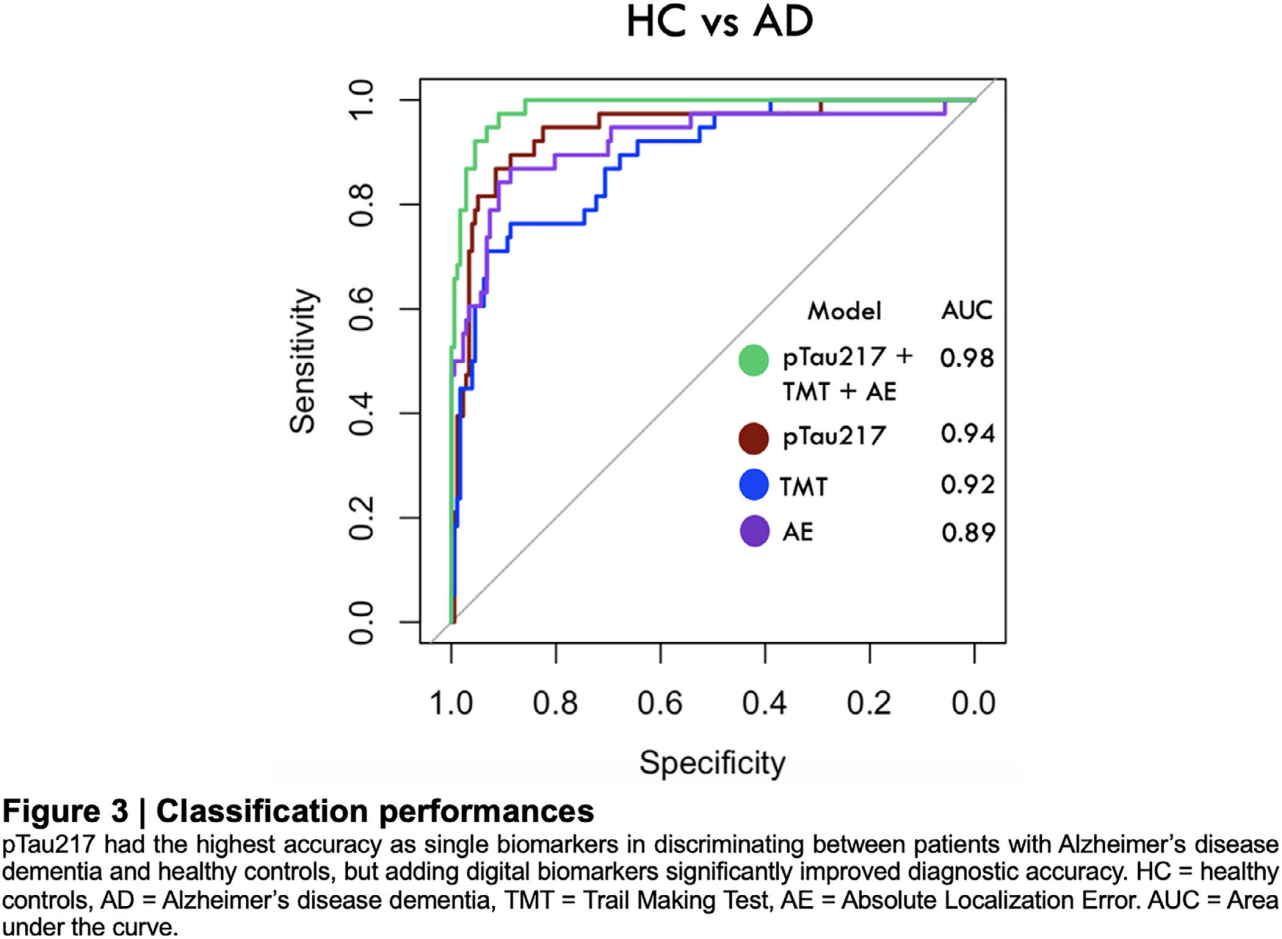# Integration of Digital and Plasma Biomarkers in Alzheimer's disease: Early Detection and Specificity Compared to Other Dementias

**DOI:** 10.1002/alz70856_097032

**Published:** 2025-12-24

**Authors:** Sofia Toniolo, Sijia Zhao, John Broulidakis, Claudia Gendarini, Dishaa Sinha, Nishat Tahira, Anna Scholcz, Benazir Amein, Andrew Fower, Cornelia M Van Duijn, Sian Thompson, Sanjay Manohar, Ivan Koychev, Masud Husain

**Affiliations:** ^1^ University of Oxford, Oxford, United Kingdom; ^2^ Università degli Studi di Milano, Milan, Italy; ^3^ University of Oxford, Oxford, Oxfordshire, United Kingdom; ^4^ Oxford University Hospitals NHS Foundation Trust, Oxford, United Kingdom

## Abstract

**Background:**

Plasma biomarkers can detect the presence of Alzheimer's disease (AD) when cognitive symptoms have not yet emerged. However, measuring cognitive function remains essential for large scale population screening, monitoring of disease progression and response to treatment. Despite the availability of several digital platforms, data on sensitivity and specificity of online testing in discriminating between different forms of dementia is currently lacking.

**Method:**

391 participants (31 subjective cognitive decline, 28 mild cognitive impairment, 88 Alzheimer's disease dementia, 18 Lewy body disease, 9 Corticobasal syndrome, 17 Frontotemporal dementia and 201 age‐matched controls) were recruited from the Oxford Centre for Cognitive Disorders and other memory centres across the UK taking part in the FAST study. They were tested on a fully remote online cognitive assessment tool, the Oxford Cognitive Testing Portal (OCTAL: https://octalportal.com), including a visual short‐term memory (Oxford Memory Test, OMT) and Trail Making Task (TMT); see Figure 1 for task schematics and metrics’ description. Plasma pTau217 was measured using the Alamar platform.

**Result:**

OCTAL's metrics captured different stages of the disease as well as discriminated between different forms of dementia (Figure 2). Identification accuracy on OMT was lower in patients with MCI as well as other primary dementias compared to controls. Absolute Localization Error could also distinguish between MCI and AD dementia patients. Localization time was significantly higher only in patients with AD and not other forms of dementia. Trail making test performance was lower in Lewy body disease patients compared to all other groups. pTau217 had the highest accuracy as single biomarker in discriminating between AD dementia and healthy controls, but adding digital biomarkers significantly improved diagnostic accuracy (Z = ‐2.0884, *p*‐value = 0.037), (Figure 3).

**Conclusion:**

These findings highlight the potential of the OCTAL platform for sensitive, and specific stratification of patients’ performance in a typical memory clinic population. They also emphasize the utility of *combining* plasma biomarkers and digital cognitive tests to improve diagnostic accuracy in AD. This fully remote platform provides a scalable approach for screening, clinical stratification and monitoring of patients with AD and other dementias.